# Associations of Social Engagement and Perceived Interpersonal Connectedness With Cognitive, Mental, and Physical Health in American Indian and Alaska Native Adults

**DOI:** 10.1177/08982643251381438

**Published:** 2025-09-21

**Authors:** Jiahui Dai, Andrew Thais, Yuxi Shi, Wenjun Fan, Erin M. Poole, Spero M. Manson, Luohua Jiang

**Affiliations:** 1 Department of Epidemiology & Biostatistics, Joe C. Wen School of Population & Public Health, Susan and Henry Samueli College of Health Sciences, 8788 University of California, Irvine, CA, USA; 2 School of Nursing, 6572 University of Pennsylvania, Philadelphia, PA, USA; 3 Centers for American Indian and Alaska Native Health, Colorado School of Public Health, 129263 University of Colorado Anschutz Medical Campus, Aurora, CO, USA

**Keywords:** social engagement, interpersonal connectedness, AD8 scores, cognitive impairment, distress, mental health, physical health

## Abstract

**Objectives:**

We examined associations of social engagement and interpersonal connectedness with cognitive function, distress, and mental and physical health in middle-aged and older American Indian and Alaska Native (AI/AN) adults.

**Methods:**

Data from 552 AI/ANs aged 55+ in the Rocky Mountain region (2019–2023). Social engagement and interpersonal connectedness were assessed using validated scales. Outcomes included cognitive impairment (adapted AD8), distress (K6 scale), and self-reported mental and physical health. Poisson regression models examined associations.

**Results:**

Greater social engagement was significantly associated with a lower risk of cognitive impairment, lower odds of suboptimal mental, and physical health among AI/AN females, while greater interpersonal connectedness was significantly associated with lower risk of cognitive impairment and reduced odds of the other outcomes among AI/AN males. Associations were stronger in urban than rural areas.

**Discussion:**

Culturally tailored interventions that promote social connectedness may enhance well-being in this underserved population. Further research on sex and residence differences is warranted.

## Introduction

Cognitive impairment has been identified as a precursor to Alzheimer’s disease and related dementias (ADRD), with its prevalence ranging from 6.7% among individuals aged 60–64 years to 25.2% for 80–84 age groups in the U.S. (Petersen et al., 2018). Cognitive impairment refers to problems with memory, attention, or decision-making that go beyond normal aging and may affect daily functioning. ADRD are more severe, progressive forms of cognitive impairment that significantly interfere with independence and functioning. While not all cognitive impairment progresses to ADRD, mild cognitive impairment (MCI) can be an early stage in the disease continuum (Organization, 2017). American Indians and Alaska Natives (AI/ANs), an underserved population projected to nearly triple by 2060 (Ortman et al., 2014), had an over 3-fold higher prevalence of cognitive impairment (54%) among older adults compared to non-Hispanic White (White) adults in 2017–2019 (Suchy-Dicey et al., 2024). This disparity highlights the importance of investigating contributing risk factors for cognitive impairment among AI/AN adults.

According to the 2024 Lancet report, in addition to well-recognized factors such as midlife diabetes and hypertension, social isolation in late life has been identified as another important risk for ADRD (Livingston et al., 2024). AI/ANs face formidable disparities in many risk factors or predictors that were reported in a recent study for ADRD or cognitive impairment (Ports et al., 2025). For instance, AI/AN adults exhibited nearly twice the prevalence of diabetes (NDSR, 2024) and higher rates of obesity, hypertension, smoking, and binge drinking compared to White adults (Breathett et al., 2020; Emert & Giano, 2023; Zhao, 2022). Moreover, AI/AN individuals often experience these health issues at an earlier age than the general U.S. population (Dai et al., 2024; Dai et al., 2025; OMH, 2022). Furthermore, they face limited access to healthcare services and timely diagnosis of cognitive impairment or ADRD due to underfunding and a shortage of clinical specialists (Blue Bird Jernigan et al., 2020), particularly among those residing in rural areas (Office, 2018). Historical trauma, mistrust, and cultural barriers further reduce engagement with national healthcare delivery systems. Consequently, the burden of cognitive impairment and ADRD is expected to be high among AI/AN adults, which presents a considerable challenge for AI/AN communities and the U.S. healthcare system.

Social health is a comprehensive term encompassing individuals’ social engagement and their sense of belonging with others or their community (McHugh et al., 2017). Poor social health is considered a potential risk factor affecting overall cognitive and physical well-being. It has been associated with an increased risk of all-cause mortality, indirectly associated with smoking, excessive alcohol consumption, and cardiovascular diseases (CVDs) (Elovainio et al., 2017; Holt-Lunstad et al., 2015; Kelly et al., 2018). Additionally, several systematic reviews have demonstrated that more social engagement is linked to improved cognitive functions, while less social engagement or a lack of interaction is associated with increased risks of cognitive decline and ADRD among older populations (Kelly et al., 2017; Kuiper et al., 2015, 2016; Penninkilampi et al., 2018; Sommerlad et al., 2023). However, to date, only a few studies have examined the associations between social health and cognitive health among AI/AN adults (Adamsen et al., 2021; Conte et al., 2015; Nelson et al., 2013). AI/AN adults have unique historical, cultural, and social experiences that may shape the relationship between social and cognitive health. Factors such as historical trauma, socioeconomic inequities, and limited access to care can heighten risks, while cultural strengths, like extended family networks and community cohesion, may offer protection. Further understanding these relationships under their distinct experiences is essential for developing culturally appropriate strategies to support cognitive health in AI/AN communities.

Mental and physical health outcomes in later life can be affected by the interactions of biological, behavioral, lifestyle, and social factors (Goins & Pilkerton, 2010). In AI/AN communities, older adults also serve as knowledge-holders and cultural caregivers, which plays an important role in preserving cultural identity and community cohesion (Viscogliosi et al., 2020). The enduring effects of colonization, including widespread violence, forced displacement, and cultural suppression, continue to shape health outcomes (Hartmann et al., 2019). These experiences have contributed to historical trauma, which manifests as persistent emotional, psychological, and physical health challenges within these communities (Brave Heart, 1998; Hartmann et al., 2019; John-Henderson et al., 2023). Within this context, social health is a fundamental aspect of the history and culture of AI/ANs, playing a crucial role in promoting both mental and physical well-being in this population (Rodning, 2019). However, establishing social connections within their own communities can be particularly challenging for AI/ANs who live in urban areas due to geographic dispersion. As of 2021, over 87% of AI/ANs resided outside their Tribal reservations (Bureau, 2021). Although 2019 U.S. national data reported that AI/ANs reported the lowest rates of depression (SAMHSA, 2019), age-adjusted rates of suicide among this population (0.014%) were higher than those of other racial/ethnic groups (CDC, 2022). Although several studies have explored social network and mental health among AI/AN youth and females (Dickerson et al., 2024; Ivanich et al., 2022; McKinley et al., 2021; Philip et al., 2016), associations of interpersonal connectedness with cognitive impairment and mental and physical well-being in middle-aged and older AI/AN adults remain unclear.

In this study, we measured social engagement, which generally reflects the nature and extent of behaviors characterized by active interactions with others, and perceived interpersonal connectedness (hereafter referred to as “interpersonal connectedness”), which captures the psychological perception of identity and belonging in terms of one’s self-concept or sense of personhood. The purpose of this study was to examine the associations of social engagement and interpersonal connectedness with cognitive impairment, distress, and mental and physical health among a sample of community volunteers of middle-aged and older AI/AN adults.

## Methods

### Data Source and Study Population

We conducted an anonymous, cross-sectional survey among urban and rural AI/AN volunteers recruited from the Rocky Mountain area (Denver, CO) from 2019 to 2023, using a community-based participatory research approach in which local community stakeholders were actively involved in project planning. Eligible AI/AN study participants represent a sample of convenience recruited through a variety of social gatherings in greater Denver and Intermountain Rockies, such as pow wows and health fairs, which were held off-reservation in both urban and rural area. A total of 552 participants ≥55 years of age were included in this investigation. The Colorado Multiple Institutional Review Board reviewed and approved the human subject protection measures. Participants underwent informed consent and were compensated $20 for completing the survey.

### Social Engagement Measurements

We assessed social engagement using a 5-item scale comprising the following questions: “In the past 12 months, how often have you: (1) attended religious and/or ceremonial services: (2) attended community activities and/or meetings; (3) volunteered to help others; (4) interacted socially with friends or relatives, and (5) interacted socially with neighbors?” Responses were recorded on a four-point scale: “1-Never”, “2-Rarely”, “3-Sometimes”, and “4-Often.” We used the mean score across these five items to represent each individual’s level of social engagement, with higher scores indicating greater social engagement.

### Interpersonal Connectedness Measurements

Our questionnaire measuring interpersonal connectedness was based on the Inclusion of Community in Self (ICS) scale (Mashek et al., 2007), which is an adaptation of the Inclusion of Other in Self Scale (Aron et al., 1992). The ICS scale has demonstrated acceptable test-retest reliability (Mashek et al., 2006). We intentionally retained the definition of “community at large” vague to encourage participants’ personalized interpretations based on their individual experiences and perspectives. Participants were asked to choose one of six pairs of overlapping circles to represent their perceived identification with their reference community. Respondents were instructed to “Circle the picture that best describes your relationship with the community at large.” More overlap presumably reflects a stronger perceived interpersonal connection to the community (Supplemental Figure 1).

### Outcomes Measurements

#### Measuring Cognitive Impairment: The Ascertain Dementia 8-Item Informant Questionnaire (AD8) and Its Adaptation for Use with AI/ANs

The AD8 is a brief, widely used questionnaire developed and validated through longitudinal studies of memory and aging to assess the likely presence of dementia or cognitive impairment (Hendry et al., 2019; Tanwani et al., 2023). It was originally developed based on the Clinical Dementia Rating (CDR): a well-established informant-based scale widely utilized in clinical research to aid in the diagnosis of dementia (Galvin et al., 2005). The AD8 has been subsequently validated for self-administration (Galvin et al., 2007), making it particularly useful in contexts where reliable informants are unavailable or when there are limited caregiver resources in clinical settings. The AD8 assesses perception of the focal adult’s problems with respect to memory, problem-solving abilities, orientation, and daily activities due to cognitive changes over the last “several years.” The questionnaire is comprised of 8 items which the respondent endorses as Yes (1) or No (0). Item-level scores are summed, resulting in a total score ranging from 0 to 8 which was the primary outcome of the present study. The AD8 is highly correlated with gold standard evaluations including the CDR14, neuropsychological testing (Morris et al., 2020), imaging, and cerebrospinal fluid biomarkers of AD (Galvin et al., 2010). A recent systematic review and meta-analysis reported that the sensitivity and specificity of the self-administered AD8 for detecting dementia were 82% and 75%, respectively (Tanwani et al., 2023). 

However, considering that the original AD8 included terms and phrasing unfamiliar or confusing to older AI/AN adults, our Community Advisory Board (CAB), whose members were selected from trusted AI/AN advocates, their family members, and health providers in AI/AN communities with prior collaborations with the research team, conducted an initial review and immediately suggested that various aspects warranted closer attention. Specifically, they encouraged modifications of the instructional set, response labels, item word choice, grammatical structure, and examples to optimize older AI/AN adults’ understanding, perceived relevance, and acceptance of the measure. We therefore undertook a careful, systematic adaptation of the AD8 along these lines, employing multiple iterative exchanges with CAB members, which began with a global discussion of the purpose of the AD8, and then solicited individual and group recommendations, reactions, and further revisions to item content and form. Supplemental Figure 2 presents side-by-side the original AD8 (left) and final version (right) adapted for our use.

In this study, the total AD8 score for each participant was determined by counting the total number of “yes” responses on the adapted AD8 questionnaires, yielding a range of possible AD8 scores from 0 to 8. The suggested cut-off score for identifying dementia or cognitive impairment is 2 or greater (Hendry et al., 2019). Therefore, individuals with higher total AD8 scores are more likely to have cognitive impairment or poorer cognitive function.

#### Distress

The 6-item Kessler Psychological Distress Scale (K6 scale), developed to assess the frequency of non-specific psychological distress, was used to measure participants’ distress in our study.Participants rated how often they experienced various feelings during the past 30 days, including nervousness, hopelessness, restlessness/fidgetiness, depression that nothing could cheer up, everything being an effort, and worthlessness. Responses were recorded “0-None of the time,” “1-A little of the time,” “2-Some of the time,” “3-Most of the time,” and “4-All of the time.” The mean score of these six items, ranging from 0 to 4 (with total scores ranging from 0 to 24), was calculated, with higher scores indicating more severe mental distress. Individuals without distress were defined as participants with a mean score on the K6 scale less than 2.17 (i.e., 13 divided by 6). The total score of 13 represents the established thresholds used in prior research to identify individuals experiencing severe mental distress (Kessler et al., 1996). Conversely, individuals reporting distress were identified as those with a mean K6 score of 2.17 or higher. The K6 scale demonstrates outstanding internal consistency and reliability with a Cronbach’s alpha of 0.87 in our study sample.

#### Mental and Physical Health Ratings

Participants’ mental and physical health were assessed using self-reported ratings on a four-point Likert scale (Fayers & Sprangers, 2002). For mental health, participants responded to the question: “In general, how would you rate your mental health, including your mood and your ability to think?” For physical health, the question posed was: “In general, how would you rate your physical health?” The response options for mental or physical health assessments included “1-Excellent,” “2-Very good,” “3-Good,” and “4-Fair.” Individuals with ratings of “3-Good” or “4-Fair” were classified as having suboptimal mental or physical health, based on prior literature suggesting that self-rated health responses of “Good,” “Fair,” and “Poor” ratings are associated with an increased risk of mortality (Schnittker & Bacak, 2014). In contrast, those with ratings of “1-Excellent” or “2-Very good” were defined as having optimal mental or physical health.

To ensure relevance and appropriateness, we selected measures previously used in a large scale, 5-year, National Institutes of Health (NIH) sponsored study of psychiatric epidemiology among American Indians recruited from two of the three largest tribes in the U.S. (Beals et al., 2005; Manson et al., 2005). These measures were rigorously evaluated and conservatively adapted for local application (Beals et al., 2003). All measures exhibited good to excellent performance characteristics.

### Statistical Analysis

Distributions of sociodemographic characteristics, social engagement, interpersonal connectedness, cognitive impairment status, distress, mental and physical health conditions, and comorbidities are presented for the study sample and subgroups stratified by sex and residence area. Means and standard deviations were calculated for continuous variables, while frequencies and percentages were reported for categorical variables. Two sample t-tests for continuous variables and chi-square tests for categorical variables were conducted to compare subpopulations. Poisson regression was applied to model the associations of social engagement and interpersonal connectedness with AD8 scores, stratified by sex and residence area. Logistic regression was used to assess the associations with distress as well as suboptimal mental and physical health, also stratified by sex and residence area. Unadjusted and adjusted associations were examined, with adjusted models including covariates of age, sex, education level, employment status, marital status, and residence area.

Additionally, we conducted a sensitivity analysis by treating distress, mental health, and physical health as continuous variables. We then applied linear regression models to assess the associations of social engagement and interpersonal connectedness with these health outcomes. The same covariates were adjusted for in this analysis as those in the logistic regression models. Lastly, Spearman correlation coefficients among the key variables were computed. All statistical analyses were performed using SAS version 9.4 (SAS, Inc. Cary, NC), and *p* values <0.05 were considered statistically significant.

## Results

The study sample had a mean age of 66.6 ± 7.4 years, with 69.0% identifying as female and 64.5% residing in urban areas. Over one-third of our sample had cognitive impairment as defined by AD8 score (33.6%) or suboptimal mental health (38.8%), while approximately half of the cohort reported suboptimal physical health (56.2%). Additionally, nearly half of the participants (48.3%) attained an education level of some college or higher. After stratifying by sex, females exhibited greater social engagement than males (3.2 vs. 3.0). However, a higher proportion of females reported suboptimal mental health compared to males (41.5% vs. 32.5%). Males were more likely to be married or living with partners (54.4% vs. 34.5%) than females. Compared to male participants, lower proportions of female participants reported heart disease (14.8% vs. 25.7%), stroke (5.1% vs. 10.8%), and alcohol use disorder (4.8% vs. 11.2%), while a higher proportion were obese (35.7% vs. 21.7%). After stratifying by residence area, we found that individuals residing in urban areas were older than those in rural areas (67.6 vs. 64.7 years). Rural adults had a higher proportion of being married or living with partners than those residing in urban areas (42.8% vs. 39.7%). Furthermore, a higher proportion of individuals in rural areas were employed (43.5% vs. 30.9%), whereas a higher proportion of those in urban areas were retired (56.2% vs. 31.1%) (all *p* values <0.05) (Table 1).

**Table 1.** Participants characteristics by sex and residence area (*N* = 552)

Characteristics	All *N* = 552	Male *N* = 171 (31.0%)	Female *N* = 381 (69.0%)	Urban *N* = 356 (64.5%)	Rural *N* = 196 (35.5%)
	Mean (SD)	Mean (SD)	Mean (SD)	Mean (SD)	Mean (SD)
Age	66.6 (7.4)	65.3 (6.6)	67.1 (7.8)^c^	67.6 (7.3)	64.7 (7.3)^d^
Social engagement^a^	3.1 (0.6)	3.0 (0.7)	3.2 (0.6)^c^	3.1 (0.6)	3.2 (0.6)
Interpersonal connectedness^b^	3.8 (1.6)	3.7 (1.7)	3.8 (1.5)	3.7 (1.6)	3.9 (1.5)
	*N* (%)	*N* (%)	*N* (%)	*N* (%)	*N* (%)
Cognitive impairment	180 (33.6)	49 (29.9)	131 (35.2)	106 (30.8)	74 (38.5)
Distress	28 (5.2)	8 (4.8)	20 (5.4)	17 (4.9)	11 (5.8)
Suboptimal mental health	205 (38.8)	53 (32.5)	152 (41.5)^c^	131 (38.3)	74 (39.6)
Suboptimal physical health	293 (56.2)	81 (50.3)	212 (58.9)	190 (57.2)	103 (54.5)
Sex					
Female	381 (69.0)	-	-	251 (70.5)	130 (66.3)
Marital status--N (%)					
Married or living with partner	219 (40.8)	92 (54.4)	127 (34.5)^c^	136 (39.7)	83 (42.8)^d^
Divorced or separated or widowed	260 (48.4)	53 (31.4)	207 (56.3)^c^	177 (51.6)	83 (42.8)^d^
Never married	58 (10.8)	24 (14.2)	34 (9.2)^c^	30 (8.8)	28 (14.4)^d^
Education--*N* (%)					
< High school graduate	58 (10.7)	16 (9.4)	42 (11.3)	33 (9.5)	25 (12.8)
High school graduate/GED	128 (23.6)	45 (26.5)	83 (22.3)	80 (23.1)	48 (24.5)
Vocational or associate’s degree	94 (17.3)	32 (18.8)	62 (16.7)	59 (17.1)	35 (17.9)
Some college	146 (26.9)	36 (21.2)	110 (29.6)	96 (27.8)	50 (25.5)
College graduate or postgraduate	116 (21.4)	41 (24.1)	75 (20.2)	78 (22.5)	38 (19.4)
Employment					
Employed	187 (35.6)	62 (38.5)	125 (34.3)	103 (30.9)	84 (43.5)^d^
Unemployed/Unable to work	92 (17.5)	26 (16.2)	66 (18.1)	43 (12.9)	49 (25.4)^d^
Retired	247 (47.0)	73 (45.3)	174 (47.7)	187 (56.2)	60 (31.1)^d^
Residence--*N* (%)					
Urban	356 (64.5)	105 (61.4)	251 (65.9)	-	-
Rural	196 (35.5)	66 (38.6)	130 (34.1)	-	-
Comorbidities--*N* (%)					
Diabetes	256 (49.6)	77 (48.1)	179 (50.3)	162 (48.4)	94 (51.9)
Hypertension	169 (35.2)	52 (35.4)	117 (35.1)	107 (34.0)	62 (37.6)
Heart disease	86 (18.2)	38 (25.7)	48 (14.8)^c^	55 (17.8)	31 (19.0)
Stroke	31 (6.8)	15 (10.8)	16 (5.1)^c^	20 (6.6)	11 (7.2)
Head Injury	43 (9.5)	15 (10.9)	28 (8.9)	29 (9.6)	14 (9.2)
Alcohol use disorder	31 (6.8)	16 (11.2)	15 (4.8)^c^	20 (6.6)	11 (7.1)
Obesity	149 (31.4)	31 (21.7)	118 (35.7)^c^	103 (32.4)	46 (29.5)

^a^Higher mean score, more social engagement.

^b^Range 1-6, higher scores indicate more overlap, reflecting higher perceived level of interpersonal connectedness.

^c^*p* < 0.05, indicates that there is a significant difference between AI/AN females and males.

^d^*p* < 0.05 indicates that there is a significant difference between urban and rural residence areas.

*Note. n* = 15 participants have missing information on marital status; *n* = 10 participants have missing information on education level; *n* = 26 participants have missing information on employment status; *n* = 12 participants have missing information on comorbidities.

Table 2 presents the unadjusted regression analysis of social engagement and interpersonal connectedness on AD8 scores, distress, and mental and physical health. Greater social engagement and interpersonal connectedness were significantly associated with lower risks of adverse cognitive and physical health outcomes. After stratifying by sex, greater social engagement was significantly associated with a lower risk of cognitive impairment [incidence rate ratio (IRR) = 0.73, 95% confidence interval (CI): 0.64–0.84], lower odds of suboptimal mental health [odds ratio (OR) = 0.60, 95% CI: 0.42–0.86] and suboptimal physical health (OR = 0.51, 95% CI: 0.35–0.74) among females but not males. Conversely, greater interpersonal connectedness was significantly associated with a lower risk of cognitive impairment (IRR = 0.90, 95% CI: 0.83–0.97) and lower odds of suboptimal mental (OR = 0.77, 95% CI: 0.62–0.95) and suboptimal physical health (OR = 0.70, 95% CI: 0.57–0.87) among males but not females. Furthermore, the associations of social engagement and interpersonal connectedness with these health outcomes were stronger among urban participants than those in rural areas.

**Table 2.** Unadjusted regression models of associations of social engagement and perceived interpersonal connectedness with health outcomes, stratified by sex and residence area

	AD8 scores^a^	Distress	Suboptimal mental health	Suboptimal physical health
	IRR (95% CI)^b^	OR (95% CI)^c^	OR (95% CI)^c^	OR (95% CI)^c^
All				
Social engagement^d^	0.82 (0.73, 0.91)^*^	0.54 (0.31, 0.95)^*^	0.65 (0.49, 0.85)^*^	0.61 (0.45, 0.81)^*^
Interpersonal connectedness^e^	0.95 (0.90, 0.99)^*^	0.85 (0.66, 1.11)	0.90 (0.80, 1.01)	0.82 (0.73, 0.92)^*^
Female				
Social engagement^d^	0.73 (0.64, 0.84)^*^	0.54 (0.26, 1.13)	0.60 (0.42, 0.86)^*^	0.51 (0.35, 0.74)^*^
Interpersonal connectedness^e^	0.97 (0.92, 1.03)	0.95 (0.70, 1.30)	0.96 (0.84, 1.11)	0.88 (0.76, 1.01)
Male				
Social engagement^d^	0.98 (0.80, 1.19)	0.50 (0.20, 1.26)	0.66 (0.40, 1.08)	0.72 (0.45, 1.15)
Interpersonal connectedness^e^	0.90 (0.83, 0.97)^*^	0.66 (0.40, 1.10)	0.77 (0.62, 0.95)^*^	0.70 (0.57, 0.87)^*^
Rural				
Social engagement^d^	0.85 (0.71, 1.02)	0.72 (0.27, 1.89)	0.83 (0.50, 1.36)	0.69 (0.42, 1.15)
Interpersonal connectedness^e^	1.00 (0.92, 1.07)	0.89 (0.57, 1.38)	0.92 (0.75, 1.13)	0.82 (0.67, 1.01)
Urban				
Social engagement^d^	0.78 (0.68, 0.90)^***^	0.45 (0.22, 0.92)^**^	0.57 (0.40, 0.81)^***^	0.57 (0.40, 0.82)^***^
Interpersonal connectedness^e^	0.91 (0.85, 0.96)^***^	0.84 (0.61, 1.15)	0.88 (0.76, 1.02)	0.81 (0.70, 0.94)^***^

^a^Higher AD8 scores indicate higher level of cognitive impairment.

^b^Results were from Poisson regression.

^c^Results were from logistic regression.

^d^Higher mean score, more social engagement.

^e^Range 1-6, higher scores indicate more overlap, reflecting higher perceived level of interpersonal connectedness.

Abbreviations: IRR: incident rate ratio; CI: confidence interval; OR: odds ratio.

^*^p < 0.05 indicates statistical significance.

**Figure 1.**
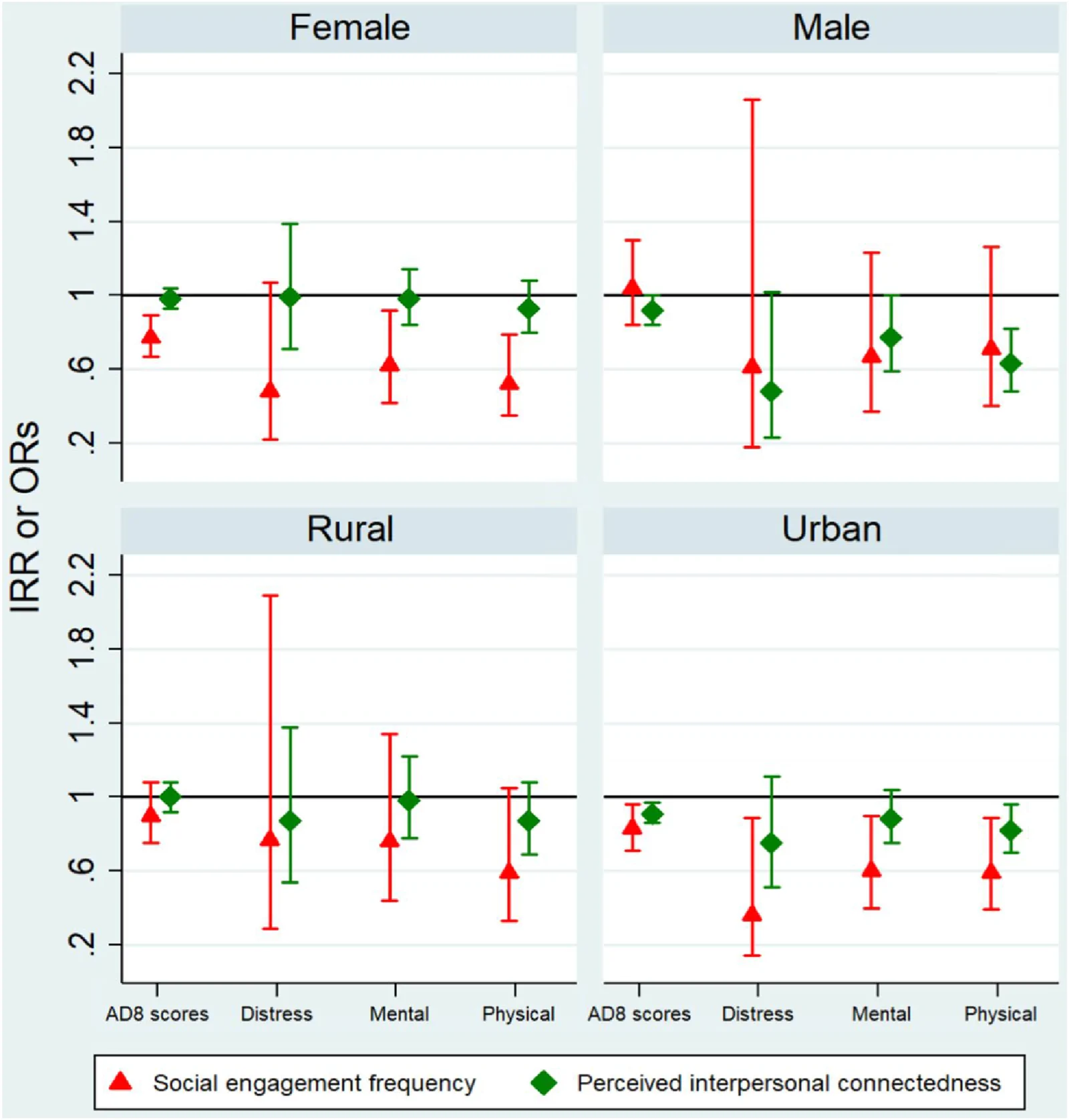
Adjusted Regression Models of Associations of Social Engagement and Perceived Interpersonal Connectedness With Health Outcomes, Stratified by Sex and Residence Area. *Note.* Adjusted models include age, sex, education, employment, marital status, and residence areas; the mean score of AD8 was used to define social engagement frequency, with higher scores indicating more social engagement. Interpersonal connectedness was measured on a scale of 1 to 6, with higher scores indicating higher perceived lever of interpersonal connectedness. IRR is from Poisson regression to model the associations of social engagement and perceived interpersonal connectedness with AD8 scores; ORs are from logistic regression to examine the associations of social engagement and perceived interpersonal connectedness with distress, mental health, and physical health. Abbreviations: IRR: incident rate ratio; ORs: odds ratios; Mental: suboptimal mental health; Physical: suboptimal physical health

Figure 1 and supplemental Table 1 present the adjusted regression models assessing the associations of social engagement and interpersonal connectedness with these four health outcomes. Overall, higher levels of social engagement were significantly associated with lower AD8 scores (IRR = 0.85, 95% CI: 0.76–0.95), lower odds of distress (OR = 0.53, 95% CI: 0.28–1.00), suboptimal mental health (OR = 0.66, 95% CI: 0.48–0.91), and suboptimal physical health (OR = 0.61, 95% CI: 0.44–0.84). In contrast, no significant association was found between interpersonal connectedness and these health outcomes, except for suboptimal physical health (OR = 0.85, 95% CI: 0.75–0.96). Stratified by sex, greater social engagement was significantly associated with a lower risk of cognitive impairment (IRR = 0.77, 95% CI: 0.67–0.89), lower odds of suboptimal mental health (OR = 0.62, 95% CI: 0.42–0.92), and suboptimal physical health (OR = 0.52, 95% CI: 0.35–0.79) among females. Conversely, among males, social engagement was not significantly associated with any health outcome. Among males, greater interpersonal connectedness was significantly associated with a lower risk of cognitive impairment (IRR = 0.92, 95% CI: 0.84–1.00) and lower odds of suboptimal mental health (OR = 0.77, 95% CI: 0.59–1.00), and suboptimal physical health (OR = 0.63, 95% CI: 0.48–0.82), while no such significant associations were observed among females. After stratifying by area of residence, among urban residents, greater social engagement and interpersonal connectedness were significantly associated with lower AD8 scores (IRR = 0.83 vs. IRR = 0.91) and lower odds of suboptimal physical health (OR = 0.59 vs. OR = 0.82). However, no significant associations of this nature were observed among rural residents.

Supplemental Tables 2 and 3 display the results of unadjusted and adjusted linear regression models examining the associations of social engagement and interpersonal connectedness with distress, mental health, and physical health. Consistent with the results from logistic regression models, after adjusting for covariates, greater social engagement was significantly associated with lower distress (β = −0.23), suboptimal mental health (β = −0.21), and suboptimal physical health (β = −0.24) among females but not males. In contrast, greater interpersonal connectedness was significantly associated with lower distress (β = −0.10) and suboptimal physical health (β = −0.17) among males but not females. Additionally, stronger associations were observed among participants residing in urban areas than those in rural areas (supplemental Table 3). Supplemental Table 4 presents the Spearman correlation coefficients among the key variables. The strongest correlation was observed between social engagement and interpersonal connectedness, with a coefficient of 0.42, indicating a moderate relationship.

## Discussion

Social engagement is generally expected to be related to health because it may promote healthier behaviors and reduce stress. Sex differences in these associations may reflect the cultural context of elderhood and the differing cultural roles of men and women in AI/AN communities. Overall, greater social engagement was significantly associated with a lower risk of cognitive impairment, psychological distress, suboptimal mental health, and suboptimal physical health after adjusting for demographic factors. When stratified by sex, we observed differing patterns: among females, greater social engagement, but not interpersonal connectedness, was significantly associated with lower likelihood of these health problems, whereas among males, greater interpersonal connectedness showed a significant association with these outcomes. Furthermore, these associations were stronger among respondents residing in urban areas compared to those in rural areas, suggesting that contextual factors, such as urbanicity may modify the health benefits of social relationships.

Our results align with previous systematic reviews that reported significant associations between greater social engagement and a lower risk of ADRD or cognitive decline among older adults (Kuiper et al., 2015, 2016; Penninkilampi et al., 2018). Specifically, our findings are consistent with a prior study indicating that older AI/AN adults with low social engagement were more likely to report self-identified memory issues (Adamsen et al., 2021). Previous research indicated that better social health was associated with a lower risk of cognitive impairment or dementia (Fancourt et al., 2020; Li et al., 2020). However, the definitions of social health adopted in those studies, namely, community engagement or participation, align more closely with our measure of social engagement, which evaluates the frequency of attending certain events, such as community activities and religious gatherings. This alignment supports our conclusions regarding the associations between social engagement and cognitive health. Additionally, our findings corroborate previous research suggesting that higher levels of social engagement are associated with a lower risk of self-reported mental and physical health issues among AI/AN adults aged 50 years and older (Nelson et al., 2013).

Several mechanisms have been proposed to explain the associations between social engagement and cognitive health. One pathway involves; CVDs limited social engagement has been found to be associated with CVDs, which in turn elevate the risk for neurodegenerative disease (Fratiglioni et al., 2004). Another proposed mechanism is neuroendocrine dysregulation. The distress hypothesis suggests that excessive secretion of stress hormones may accelerate the degeneration of hippocampal structures, brain regions critical for memory and learning, which could adversely impact cognitive health (DeYoung et al., 2010; Sapolsky et al., 2002). Additionally, individuals suffering worse social health are more likely to experience cognitive deficits, thereby increasing their risk of developing cognitive impairment or ADRD (Evans et al., 2018; Fratiglioni et al., 2000; Miceli et al., 2019). These mechanism illustrate how worse social health may biologically contribute to developing cognitive impairment.

Although the measures of social engagement and interpersonal connectedness both assess aspects of social health, they demonstrated only a moderate correlation in our study. With respect to sex stratification, we found that greater social engagement was significantly associated with a lower risk of adverse cognitive, mental, and physical health outcomes among females but not males. Limited consideration has been given to these associations relative to sex differences among U.S. adults; however, our findings are consistent with prior studies conducted outside of America. A study of aging adults in Australia found that social isolation and low social support were associated with poorer cognitive function in females but not in males (Joyce et al., 2022). Similarly, research from Japan reported that social engagement in community activities was protective against dementia for females, but not for males (Murata et al., 2019). These differences may result from variations in sex-specific behaviors related to social engagement or participation in social activities. Social norms often encourage females to demonstrate heightened attentiveness to social relationships and to actively address the emotional needs of those around them (Erickson, 2005; Umberson et al., 2015). Consequently, females tend to establish stronger social support networks, including close friendships and connections with neighbors, and benefit from them (Taylor et al., 2002). But women’s larger, more engaged social networks may also introduce added emotional and psychological burden and concomitant stress (Kessler, 1979; Kessler et al., 1985). Conversely, societal norms often pressure males to project physical and emotional resilience, de-emphasizing close relationships and restricting opportunities for emotional expression (Rosenfield & Mouzon, 2013; Umberson et al., 2015). Meanwhile, males usually value more their contributions to their community, which is better measured by the interpersonal connectedness scale in this study. These distinctions in social norms and relationships may contribute to the observed differences in associations between social engagement and cognitive and physical well-being among females and males in our study.

Additionally, previous research has highlighted shifts in sex roles among older adults after retirement; older women often have a higher frequency of social engagement than men (Ejechi, 2015; Huang & Yang, 2013). Consistent with these findings, our study observed that AI/AN females reported slightly higher frequency of social engagement than males at a mean age of 66.6 years and therefore may have stronger social networks and support than males. Our analysis showed that a smaller proportion of females over the age of 55 were employed compared to males of the same age. For these females, social engagement may offer meaningful health benefits. On the other hand, employed males may already obtain sufficient social interaction through work, potentially reducing the additional impact of non-work-related social engagement on their health outcomes. Moreover, it is important to consider the cultural context of elderhood in AI/AN communities. In many such communities, elderhood is associated with meaningful roles, such as caregivers, cultural stewardship, and community leadership (Conte et al., 2015; Viscogliosi et al., 2020). These culturally rooted responsibilities may foster forms of social engagement that extend beyond employment and contribute positively to their health. Future research on sex differences in social engagement and cognitive and mental health, particularly among U.S. underserved populations, is warranted.

In contrast to social engagements, our study found that greater interpersonal connectedness was significantly associated with a lower risk of adverse cognitive and physical health outcomes in AI/AN males but not in females. Limited literature has examined sex difference in interpersonal connectedness and associated health outcomes in AI/AN communities, particularly as measured by the ICS scale, and cognitive or physical health outcomes. Our study provides novel insights into these associations among AI/AN adults. Prior study suggests that patterns of connectedness among AI/AN males and females are shaped, in part, by culturally specific roles and expectations. For example, historical accounts often depict AI/AN males in public roles like warriors or hunters, while females are commonly associated with domestic responsibilities (Kim et al., 2020). Therefore, today, AI/AN males with greater interpersonal connectedness may actively serve their community through mentorship, leadership in local organizations, or involvement in shaping community initiatives. These forms of community involvement among males may contribute to a heightened sense of purpose and accomplishment, potentially fostering a stronger sense of achievement compared to females. This enhanced fulfillment may, in turn, be significantly associated with improved cognitive and physical well-being in males. Further research is needed to use the ICS scale to measure interpersonal connectedness among other communities and to validate our findings regarding sex differences in the relationships between interpersonal connectedness and cognitive and physical health outcomes.

With respect to residence, greater social engagement and interpersonal connectedness were significantly associated with a lower risk of adverse cognitive and physical health outcomes among AI/ANs residing in urban areas, but not in rural areas. These findings align with previous research supporting positive associations between social engagement and mental well-being among a sample of 150 AI/ANs living in urban areas across 20 U.S. states (Dickerson et al., 2024). Social engagement and interpersonal connectedness play essential roles in promoting mental health and overall well-being within AI/AN communities while also preserving their language, culture, and societal norms (Rodning, 2019). However, geographic dispersion and lack of a critical mass of fellow community members, it can be challenging to establish and maintain social connections, especially for AI/ANs residing in urban areas (Dickerson et al., 2024). Furthermore, many AI/ANs in urban areas have faced difficulties in obtaining employment, which exacerbates feelings of isolation and discrimination, driven by a growing disconnection from their traditional culture and practices (Weaver, 2012). More social engagement and interpersonal connectedness in urban areas may therefore have a stronger influence on their cognitive and physical health. Additionally, in our study sample, approximately two-thirds of the participants resided in urban areas, the larger sample size may provide greater statistical power to detect significant associations observed among urban AI/ANs. Our findings suggest that tailored strategies aimed at strengthening social engagement and interpersonal connectedness among AI/ANs in urban areas may promote cognitive and physical well-being.

To the best of our knowledge, this is the first study to assess the associations of social engagement and interpersonal connectedness with cognitive, mental, and physical health outcomes among middle-aged and older AI/ANs. However, several limitations should be acknowledged. First, we employed a convenient, non-random sampling method to recruit participants, which may introduce selection bias, affect the accuracy of the observed associations, and limit the generalizability. Second, the study cohort was limited to AI/ANs residing in the Rocky Mountain region and had a relatively high level of education attainment, which may restrict the generalizability of the findings to the broader AI/AN population. Third, data were collected through self-report surveys without verification from medical records, making them susceptible to potential recall bias and inconsistencies in self-reported assessments. Additionally, the cross-sectional study design limits our ability to determine temporality or causality between social engagement or interpersonal connectedness and the studied outcomes. While greater social engagement and interpersonal connectedness may protect AI/ANs from cognitive impairment or poor mental and physical health, it is also plausible that poor cognitive or physical health leads to reduced social engagement and interpersonal connectedness. Lastly, the capture of interpersonal connectedness, based on our “community” definition, may be impacted by factors such as social status, humility, or cultural expression. Although this approach may introduce measurement error and make it more difficult to detect statistical effects, associations observed under these conditions may be considered more robust and reliable (Mashek et al., 2006).

In conclusion, this study suggests that higher levels of social engagement were associated with better cognitive and physical health among AI/AN female respondents ≥55 years, while greater interpersonal connectedness was linked to improvements in these outcomes among AI/AN males. These findings underscore the potential for tailored health interventions that consider historical and cultural backgrounds, as well as the important role of older adults, such as cultural stewardship or community leadership, to promote positive social and community connections and enhance cognitive and physical well-being in this population. Future research using longitudinal data promises to shed light on potential causal relationships and sex and residential differences in these associations among AI/AN adults and other populations.

## Supplemental Material

Supplemental Material - Associations of Social Engagement and Perceived Interpersonal Connectedness With Cognitive, Mental, and Physical Health in American Indian and Alaska Native Adults Supplemental Material for Associations of Social Engagement and Perceived Interpersonal Connectedness With Cognitive, Mental, and Physical Health in American Indian and Alaska Native Adults by Jiahui Dai, Andrew Thais, Yuxi Shi, Wenjun Fan, Erin M. Poole, Spero M. Manson, and Luohua Jiang in Journal of Aging and Health

## Data Availability

The study data have not been made available for sharing broadly because of Tribal regulations regarding data confidentiality and security. However, we are happy to provide the SAS codes upon request. The research center where Dr. Spero Manson, one of the multiple principal investigators (MPIs), works has developed a process to explore opportunities to share project data that would benefit the health of the AI/AN people engaged in the project.*
