# Supplementary material for: Associations of Social Engagement and Perceived Interpersonal Connectedness With Cognitive, Mental, and Physical Health in American Indian and Alaska Native Adults
Source: J Aging Health. 2025 Sep 21;38(9):564–76. doi: 10.1177/08982643251381438 (PMC13542388; doi:10.1177/08982643251381438)
Supplement: Supplementary file 1 [file sj-pdf-1-jah-10.1177_08982643251381438.pdf]

Supplementary materials

Supplemental Figure 1. The questionnaire for perceived interpersonal connectedness

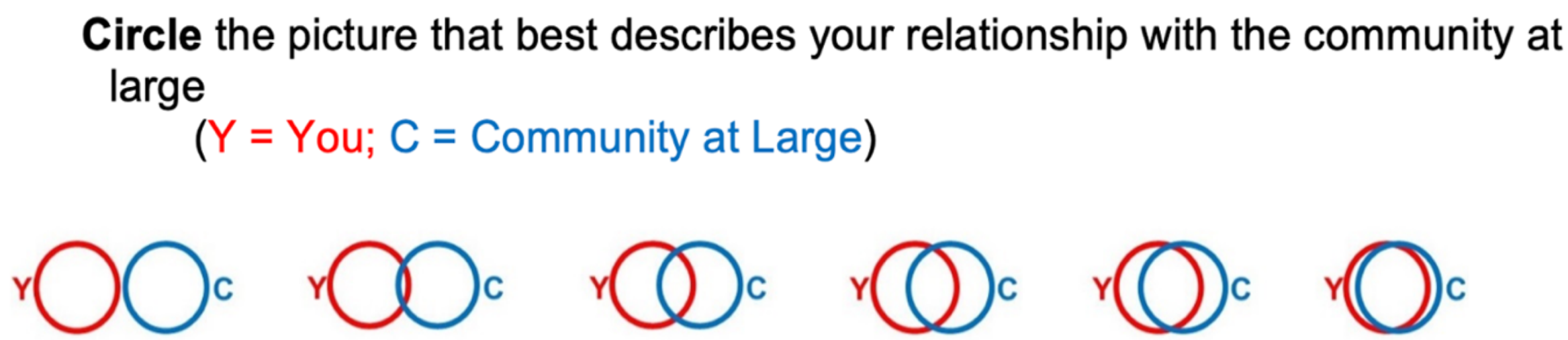

Note: This figure was adapted from the Inclusion of Community In the Self (ICS) Scale, "Mashek, D., Cannaday, L. W., & Tangney, J. P. (2007). Inclusion of community in self scale: A single-item pictorial measure of community connectedness. *Journal of community psychology*, *35*(2), 257-275".

Supplemental Figure 2. The original AD8 (left) and final version (right) adapted AD8 used in the study

Original AD8 Dementia Screening Interview

Patient ID#:__________
CS ID#:__________
Date:__________

Remember, "Yes, a change" indicates that there has been a change in the last several years caused by cognitive (thinking and memory) problems.	YES, A change	NO, No change	N/A, Don't know
**1.** Problems with judgment (e.g., problems making decisions, bad financial decisions, problems with thinking)			
**2.** Less interest in hobbies/activities			
**3.** Repeats the same things over and over (questions, stories, or statements)			
**4.** Trouble learning how to use a tool, appliance, or gadget (e.g., VCR, computer, microwave, remote control)			
**5.** Forgets correct month or year			
**6.** Trouble handling complicated financial affairs (e.g., balancing checkbook, income taxes, paying bills)			
**7.** Trouble remembering appointments			
**8.** **Daily** problems with thinking and/or memory			
**TOTAL AD8 SCORE**			

Adapted from Galvin JE et al, The AD8, a brief informant interview to detect dementia, Neurology 2005:65:559-564

Copyright 2005. The AD8 is a copyrighted instrument of the Alzheimer's Disease Research Center, Washington University, St. Louis, Missouri. All Rights Reserved.

Adapted AD8 Dementia Screening Interview

Participant ID#: ____________________
Date: ____________________

For each question below, please mark "Yes" or "No" to say if there has been a change caused by trouble with thinking and memory over the last several years.	Yes, there's been a change	No, there's been no change	I don't know
1. A hard time making decisions, making risky money decisions, or focusing your thoughts.			
2. Less interest in doing crafts or participation in cultural activities.			
3. Repeating the same stories, questions, or statements again and again.			
4. A hard time learning to use a new tool, appliance, or gadget (for example, a new phone, computer, microwave, or remote control).			
5. Forgetting what month or year it is.			
6. A hard time handling complicated money matters yourself (things like paying bills or doing taxes).			
7. A hard time remembering appointments without help.			
8. Having a hard time focusing your thoughts or remembering things on **a daily basis.**			
**TOTAL AD8 SCORE**			

Adapted from Galvin JE et al, The AD8, a brief interview to detect dementia, Neurology 2005:65:559-564

Copyright 2005. The AD8 is a copyrighted instrument of the Alzheimer's Disease Research Center, Washington University, St. Louis, Missouri. All rights reserved.

Supplemental Table 1. Adjusted regression models of associations of social engagement and perceived interpersonal connectedness with health outcomes, stratified by sex and residence area

	AD8 scores[a]	Distress	Suboptimal mental health	Suboptimal physical health
	IRR (95% CI)[b]	OR (95% CI)[c]	OR (95% CI)[c]	OR (95% CI)[c]
**All**				
Social engagement[d]	0.85 (0.76, 0.95)*	0.53 (0.28, 1.00)*	0.66 (0.48, 0.91)*	0.61 (0.44, 0.84)*
Interpersonal connectedness[e]	0.97 (0.92, 1.01)	0.84 (0.63, 1.11)	0.92 (0.81, 1.05)	0.85 (0.75, 0.96)*
**Female**				
Social engagement[d]	0.77 (0.67, 0.89)*	0.48 (0.22, 1.07)	0.62 (0.42, 0.92)*	0.52 (0.35, 0.79)*
Interpersonal connectedness[e]	0.98 (0.93, 1.04)	0.99 (0.71, 1.39)	0.98 (0.84, 1.14)	0.93 (0.80, 1.08)
**Male**				
Social engagement[d]	1.04 (0.84, 1.30)	0.61 (0.18, 2.06)	0.67 (0.37, 1.23)	0.71 (0.40, 1.26)
Interpersonal connectedness[e]	0.92 (0.84, 1.00)*	0.48 (0.23, 1.02)	0.77 (0.59, 1.00)*	0.63 (0.48, 0.82)*
**Rural**				
Social engagement[d]	0.90 (0.75, 1.08)	0.77 (0.29, 2.09)	0.76 (0.44, 1.34)	0.59 (0.33, 1.05)
Interpersonal connectedness[e]	1.00 (0.92, 1.08)	0.87 (0.54, 1.38)	0.98 (0.78, 1.22)	0.87 (0.69, 1.08)
**Urban**				
Social engagement[d]	0.83 (0.71, 0.96)*	0.36 (0.14, 0.89)*	0.60 (0.40, 0.90)*	0.59 (0.39, 0.89)*
Interpersonal connectedness[e]	0.91 (0.86, 0.97)*	0.75 (0.51, 1.11)	0.88 (0.75, 1.04)	0.82 (0.70, 0.96)*

Note: Age, sex, education, employment, marital status, and residence areas were adjusted.
[a] Higher AD8 scores indicate higher level of cognitive impairment.
[b] Results were from Poisson regression.
[c] Results were from logistic regression.
[d] Higher mean score, more social engagement.
[e] Range 1-6, higher scores indicate more overlap, reflecting higher perceived level of interpersonal connectedness.
Abbreviations: IRR: incident rate ratio; CI: confidence interval; OR: odds ratio.
*$p<0.05$ indicates statistical significance.

Supplemental Table 2. Unadjusted linear regression models of associations of social engagement and perceived interpersonal connectedness with health outcomes, stratified by sex and residence area

	K6 Scale[a]	Mental health rating[b]	Physical health rating[c]
	**Estimate (95% CI)**	**Estimate (95% CI)**	**Estimate (95% CI)**
**All**			
Social engagement[d]	-0.18 (-0.28, -0.08)*	-0.20 (-0.33, -0.07)*	-0.21 (-0.33, -0.08)*
Interpersonal connectedness[e]	-0.05 (-0.10, -0.01)*	-0.08 (-0.13, -0.03)*	-0.09 (-0.14, -0.04)*
**Female**			
Social engagement[d]	-0.23 (-0.35, -0.10)*	-0.24 (-0.40, -0.08)*	-0.27 (-0.42, -0.12)*
Interpersonal connectedness[e]	-0.04 (-0.09, 0.01)	-0.05 (-0.12, 0.01)	-0.05 (-0.11, 0.01)
**Male**			
Social engagement[d]	-0.10 (-0.28, 0.08)	-0.18 (-0.40, 0.04)	-0.14 (-0.36, 0.08)
Interpersonal connectedness[e]	-0.00 (-0.16, -0.02)*	-0.13 (-0.22, -0.05)*	-0.17 (-0.25, -0.08)*
**Rural**			
Social engagement[d]	-0.13 (-0.31, 0.05)	-0.08 (-0.32, 0.16)	-0.13 (-0.36, 0.10)
Interpersonal connectedness[e]	-0.08 (-0.15, -0.01)*	-0.10 (-0.19, -0.00)*	-0.09 (-0.18, -0.00)*
**Urban**			
Social engagement[d]	-0.21 (-0.33, -0.10)*	-0.26 (-0.42, -0.11)*	-0.24 (-0.38, -0.09)*
Interpersonal connectedness[e]	-0.05 (-0.10, 0.00)	-0.07 (-0.14, -0.01)*	-0.09 (-0.15, -0.03)*

*$p<0.05$ indicates statistical significance.
[a] Higher scores on the K6 scale indicate more distress.
[b] Higher scores on mental health ratings reflect worse mental conditions.
[c] Higher scores on physical health ratings reflect worse physical conditions.
[d] Higher mean score, more social engagement.
[e] Range 1-6, higher scores indicate more overlap, reflecting higher perceived level of interpersonal connectedness.

Supplemental Table 3. Adjusted linear regression models of associations of social engagement and perceived interpersonal connectedness with health outcomes, stratified by sex and residence area

	K6 Scale[a]	Mental health rating[b]	Physical health rating[c]
	**Estimate (95% CI)**	**Estimate (95% CI)**	**Estimate (95% CI)**
**All**			
Social engagement[d]	-0.15 (-0.26, -0.05)*	-0.17 (-0.31, -0.04)*	-0.19 (-0.32, -0.06)*
Interpersonal connectedness[e]	-0.05 (-0.09, -0.01)*	-0.06 (-0.11, 0.00)*	-0.07 (-0.12, -0.02)*
**Female**			
Social engagement[d]	-0.23 (-0.35, -0.10)*	-0.21 (-0.38, -0.05)*	-0.24 (-0.39, -0.09)*
Interpersonal connectedness[e]	-0.03 (-0.07, 0.02)	-0.04 (-0.10, 0.03)	-0.03 (-0.09, 0.03)
**Male**			
Social engagement[d]	-0.02 (-0.22, 0.17)	-0.15 (-0.40, 0.09)	-0.15 (-0.39, 0.09)
Interpersonal connectedness[e]	-0.10 (-0.18, -0.02)*	-0.10 (-0.19, 0.00)	-0.17 (-0.27, -0.08)*
**Rural**			
Social engagement[d]	-0.09 (-0.28, 0.10)	-0.07 (-0.32, 0.17)	-0.20 (-0.43, 0.04)
Interpersonal connectedness[e]	-0.06 (-0.13, 0.01)	-0.07 (-0.16, 0.03)	-0.07 (-0.16, 0.02)
**Urban**			
Social engagement[d]	-0.19 (-0.31, -0.06)*	-0.23 (-0.39, -0.06)*	-0.19 (-0.35, -0.04)*
Interpersonal connectedness[e]	-0.06 (-0.10, -0.01)*	-0.06 (-0.12, 0.01)	-0.08 (-0.14, -0.02)*

Note: age, sex, education, employment, marital status, and residence area were adjusted.
*$p<0.05$ indicates statistical significance.
[a] Higher scores on the K6 scale indicate more distress.
[b] Higher scores on mental health ratings reflect worse mental conditions.
[c] Higher scores on physical health ratings reflect worse physical conditions.
[d] Higher mean score, more social engagement.
[e] Range 1-6, higher scores indicate more overlap, reflecting higher perceived level of interpersonal connectedness.

Supplemental Table 4. Spearman correlation coefficients among the key variables

	Social engagement	Interpersonal connectedness	Age	Education level	Employment
**Social engagement[a]**	1	0.42*	-0.04	0.19*	-0.01
**Interpersonal connectedness[b]**	0.42*	1	-0.06	0.13*	-0.11*
**Age[c]**	-0.04	-0.06	1	-0.004	0.52*
**Education level[d]**	0.19*	0.13*	-0.004	1	-0.04
**Employment[e]**	-0.01	-0.11*	0.52*	-0.04	1

*$p<0.05$ indicates statistical significance.
[a] Mean score was used to define social engagement frequency; range 1-4, higher mean score, more social engagement.
[b] Range 1-6, higher scores indicate more overlap, reflecting higher perceived level of interpersonal connectedness.
[c] Age range: 55-94 years.
[d] Education level from 1= < High school graduate to 5= College graduate or postgraduate.
[e] Employment status: 1= Employed, 2= Unemployed/Unable to work, 3= Retired.
